# A Clinical Trial Design for Evaluating Topical Antimicrobials in Chronic Wounds: The BLEU Trial

**DOI:** 10.3390/life13101983

**Published:** 2023-09-28

**Authors:** Thomas Serena, Emily King, Theresa Boyer, Khristina Harrell

**Affiliations:** SerenaGroup Research Foundation, Cambridge, MA 02140, USA; eking@serenagroups.com (E.K.); serena@serenagroups.com (T.B.);

**Keywords:** chronic wounds, topical antimicrobials, clinical trial design, surrogate endpoints, fluorescence imaging

## Abstract

Chronic wound management is a global challenge. Millions of patients suffer from nonhealing ulcers and health systems are overwhelmed by the growing demand for treatment. Despite the prevalence of chronic wounds, the emergence of wound centers and specialized physicians is a recent phenomenon. Likewise, clinical research in wound healing is in its infancy. To date, many of the products in wound care have little or no clinical evidence. The field needs standardized clinical trial design, endpoints recognized by clinicians and payers, and improved overall clinical evidence. Wound healing is impeded by the presence of bacterial biofilms, which exist in most chronic wounds. It is not surprising that biofilm disruption is the focus of wound management and essential to the healing process. Multiple laboratory and preclinical studies demonstrate promising efficacy of several antimicrobials in treating biofilms; however, the field lacks in vivo clinical studies. In addition, a standardized trial design to evaluate efficacy of antimicrobials in chronic wounds does not exist. The advent of new diagnostic technologies, such as fluorescence imaging, has led to clinical trial designs that are reliable, easier to conduct, and cost efficient. The protocol presented here describes a randomized controlled double-blind trial designed to evaluate antiseptics in chronic wounds.

## 1. Introduction

Chronic wounds cause patient suffering, challenge clinicians, and stress health systems across the world. A new medical specialty focusing on hard-to-heal wounds has emerged to meet the increasing demand posed by millions of patients with wounds that fail to heal. As with any new field, there is limited evidence supporting the treatment regimens currently in use. In addition, clinical trials in wound healing lack standardization in endpoints and consistency in design. The goal of the wound care community is to increase the level of evidence for treatment modalities, standardize clinical trial design, and develop meaningful endpoints.

Several factors contribute to delayed healing in chronic wounds [1]. Key among them are excessive amounts of bacteria. A recent clinical trial demonstrated that more than 80% of chronic wounds harbor bacterial levels that impede wound healing [2]. Moreover, the bacteria in chronic wounds exist preferentially in a biofilm phenotype [3]. The bacteria in biofilms are resistant to antibiotics and may be protected from topical antiseptics [4]. They delay wound healing and frequently recur [5]. As a result, clinicians have adopted a biofilm-based approach in the treatment of chronic wounds that includes debridement, wound cleansing, and the use of topical antimicrobials [6]. Antiseptic cleansers and topical antimicrobials are supported by preclinical evidence [7], but there are few well powered prospective in vivo clinical trials.

In current practice, the choice of wound cleansers is based on professional experience, clinician preference, or institutional policy [7]. Unfortunately, there is sparse evidence that cleansers and topical antimicrobials reduce biofilm or promote healing in patients with chronic wounds. In order to garner greater evidence for these agents, a randomized controlled double blind clinical trial design is presented.

The efficacy of the treatment regimen in eliminating bacteria and biofilms is achieved using fluorescence imaging (MolecuLight, Toronto, Canada). Fluorescence imaging (FL) is a validated noninvasive point-of-care method for assessing bacterial load in chronic wounds [2]. It detects bacterial levels greater than >104 CFU/g [8] and obviates the need for invasive biopsies. Incorporating fluorescence imaging into the trial design permits rapid and repeatable assessment of bacterial levels throughout the study: This trial will assess bacterial levels weekly. This design would be impractical using tissue cultures that are invasive and expensive.

Described here is the BIAKOS (Sanara, Fort Worth, TX, USA) lower extremity ulcer trial (BLEU): a randomized double-blind controlled trial designed to compare healing rates between standard of care (normal saline wash and an amorphous gel) to the combination of synergistic antimicrobial cleanser and antimicrobial gel in chronic lower extremity ulcers. It is the first major trial on antimicrobials and one of the few double-blind studies to be conducted in the wound care space.

In two biofilm models, different types of commercially available wound cleansers will be compared: BIAKOS, a polyhexamethylene biguanide (PHMB)-based cleanser (PHMB-1), has the largest biofilm reductions when compared to all other studied cleansers after a single application and significant reductions in MRSA and C albicans biofilms following daily treatments over 72 h compared to all other test articles. Results of this preclinical trial demonstrate that a PHMB-based cleanser used daily performed significantly better than other cleansers [9]; however, studies to examine the clinical effectiveness on bacterial load and actual wound outcomes are needed.

Commercially available antimicrobials promote products based on lab or preclinical evidence rather than trials in the wound clinic population. The BIAKOS™Lower Extremity Ulcers trial is a randomized double-blind controlled trial designed to compare healing rates between normal saline wash and amorphous gel to the combination of synergistic antimicrobial cleanser and antimicrobial gel in chronic lower extremity ulcers. It is the first major trial on antimicrobials and one of the few double-blind studies to be conducted in the wound care space.

## 2. Materials and Methods

BLEU is a randomized double-blind controlled clinical trial comparing a synergistic antimicrobial cleanser (AMC) and an antimicrobial gel (AMG) to a normal saline wash and an amorphous gel (NSS-HG) in reducing bioburden and promoting healing in chronic lower extremity ulcers (clinicaltrials.gov #NCT05107050). This study will be conducted at up to five SerenaGroup^®^ Research Foundation centers throughout the United States with up to 60 subjects with acute or chronic non healing wounds participating. The study is anticipated to be completed within one year. The study population will be drawn from patients suffering from chronic wounds who are attending wound clinics or residing in skilled nursing facilities. Section 2.6 details the inclusion and exclusion criteria for the BLEU trial.

### 2.1. Objectives and Endpoints

The primary objective for the BLEU clinical trial is to compare healing rates (percent area reduction, PAR) between normal saline wash and an amorphous gel (NSS-HG) to the combination of a synergistic antimicrobial cleanser (AMC) and an antimicrobial gel (AMG) in chronic lower extremity ulcers, including diabetic foot (DFU) and venous leg ulcers (VLU). The primary endpoint is PAR in 4 weeks, with a goal healing rate of 40% in 4 weeks. There is a large body of evidence that supports this rate of healing for DFUs and VLUs [10], and the FDA-sponsored Wound Care Collaborative Community (WCCC) has recommended it as a surrogate endpoint [11].

In addition, an important endpoint in antiseptic clinical trials is a reduction in bacteria. The use of point-of-care fluorescence imaging has simplified this process. Fluorescence imaging will be obtained at every visit and the reduction in bacterial bioburden will be compared between ulcers treated with NSS-HG and AMC-AMG.

Additional secondary endpoints common to most wound-healing clinical trials will be the difference in pain scores and adverse events between subjects treated with NSS-HG and AMC-AMG.

Exploratory endpoints are encouraged if they do not interfere with the primary or secondary endpoints. They contribute to the body of knowledge on chronic wounds and can improve the conduct of future trials. This study will examine host protease levels (matrixmetaloproteases 8 and 9 and neutrophil elastase) using a point-of-care lateral flow test (WOUNDCHEK PROTEASE TEST, WoundChek, Gargrave, UK). In clinical trials, the diagnostic accurately identified wounds with high levels of inflammation. In wounds with a positive test for elevated protease activity (EPA) only 10% were healed at 12 weeks [12].

### 2.2. Blinding

In the BLEU trial, the subjects and investigators will be blinded to the treatment arm. Subjects will be randomized in 1:1 fashion to either NSS-HG or AMC-AMG. Unblinded staff will prepare the solutions for the two study arms the morning of the day of enrollment. NSS-HG or AMC-AMG will be placed in labeled containers consistent with the randomization scheme. The NSS-HG or AMC-AMG will be applied at randomization and weekly thereafter. The unblinded research staff will provide the subject with a 4-week supply of NSS-HG or AMC-AMG for home use. For this trial, the solutions will be mixed in the clinic for practical reasons. In future trials utilizing this design, the aim is to have the Investigational sites receive the product and control in generic containers identified by numbers.

Given the low risk of the device, the authors do not anticipate breaking the blind during the trial; however, if unblinding is required for a medical emergency the medical monitor will be notified immediately. The subject’s treatment assignment will not be revealed to other blinded staff unless required for safety reasons. The circumstances leading to the unblinding, and date will be fully documented in the subject’s source documentation. The subject will be withdrawn from the trial at the time of unblinding.

### 2.3. Diagnosis

One of the challenges in conducting clinical trials in wound healing is ensuring that the wound type under study has been appropriately diagnosed. The clinical trial described here examines two ulcer types: diabetic foot (DFU) and venous leg ulcerations (VLU). The diagnosis of these two wounds is primarily clinical. DFUs occur on the plantar aspect of the foot in diabetic patients with sensory neuropathy. An objective assessment of vascular status using the ankle brachial index (ABI), the toe brachial index (TBI), or another screening test will rule out patients with significant ischemia. Venous leg ulcers develop on the medial or lateral gaiter regions of the lower extremities. Most clinical trials enroll VLU patients based on clinical signs and symptoms: lower extremity edema, hyperpigmentation of the skin, loss of hair, atrophy blanch, thickening of the nails, and lip dermatosclerosis. The venous ulcer is shallow with an irregular border. For ulcers that exhibit these signs, the diagnosis is straightforward. For ulcers suspected to be VLUs but that do not have the characteristic appearance, the investigator should consider biopsy to rule out vasculitis, cancer, or other pathology. The investigator may also wish to obtain a venous duplex scan to demonstrate venous insufficiency. In the past, VLU clinical trials mandated venous duplex procedures; however, this adds considerable cost to the trial without adding significant benefit.

### 2.4. Vulnerable Populations

Although vulnerable subjects will not specifically be recruited for this study, vulnerable subjects may be present in the potential subject pool. Vulnerable subjects are defined as patients who are pregnant, fetuses/children, or prisoners. Additional procedures will not be required to ensure protection for these human subjects.

### 2.5. Standard of Care

The trial design calls for both groups to receive the standard-of-care treatment. The recognized standard of care (SOC) for diabetic foot ulcers (DFUs) is debridement, off-loading, maintaining proper moisture balance, and control of bacterial burden [13]. Reducing bacterial load and moisture balance are addressed in the protocol. The gold standard for off-loading the diabetic foot is a total contact cast (TCC) [14]; however, few clinical trials have utilized this method for a variety of reasons, including expense, difficulty in application, and patient acceptance. In antiseptic trials that require daily applications of the study agent TCCs are impractical. The alternative is the use of a fixed ankle walker; however, patient adherence has been a problem with removable walkers [15]. Recent advances in off-loading boots may improve patient adherence. Debridement is an essential procedure in the treatment of the DFU [16]. It is advisable to monitor the extent of debridement in DFU clinical trials. In this trial, debridement will be assessed through weekly digital monitoring of pre- and post-debridement photos.

The mainstay of the standard of care for VLUs is multilayer compression and routine debridement [1]. The authors recommend supplying the same compression device to all of the sites to standardize the compression. Small differences in compression devices can add heterogeneity to the trial.

### 2.6. Subject Characteristics

Patients who suffer from acute or chronic wounds will be recruited for this study from participating wound clinics. Once patients agree to adhere to the study schedule (weekly visits and follow-up regimen) and read and sign the IRB approved Informed Consent Form, screening will be conducted to determine whether the inclusion criteria were met:

- The patient must be at least 18 years old, and if female of childbearing potential, must be willing to use acceptable methods of contraception (birth control pills, barriers, or abstinence);
- The presence of a DFU extending through the dermis or subcutaneous tissue or the presence of a full-thickness VLU;
- To assess the primary endpoint of the trial, the index ulcer must have been present for longer than four weeks prior to screening;
- A DFU size between 0.75 cm^2^ and 5 cm^2^ and a VLU size between 2.0 cm^2^ and 20.0 cm^2^ at first treatment visit;
- Circulation to the affected extremity is adequate, as demonstrated by a transcutaneous oxygen measurement (TCOM) or skin perfusion pressure (SPP) measurement of ≥30 mmHg, OR an ankle brachial index between 0.7 and ≤1.3 OR a toe brachial index > 0.5 within 3 months of the first screening visit.

If any of the following exclusion criteria are met, patients will be excluded from participating in the study:

- Study ulcer(s) deemed by the investigator to be caused by a medical condition other than diabetes;
- Surgery for operative debridement or revascularization is planned for the ulcer to be treated;
- Index ulcer has a history of cancer or, in the opinion of the investigator, is suspected to be cancer and should undergo an ulcer biopsy to rule out a carcinoma of the ulcer;
- Subjects with a history of more than two weeks of treatment with immunosuppressants (including systemic corticosteroids of > 10 mg daily dose), cytotoxic chemotherapy, or application of topical steroids to the ulcer surface within one month prior to first screening visit, or who receive such medications during the screening period, or who are anticipated to require such medications during the study;
- History of radiation at the ulcer site;
- Subject who cannot adhere to strict offloading according to protocol standards, in the opinion of the investigator;
- Presence of any condition(s) that seriously compromise(s) the subject’s ability to complete this study or has a known history of poor adherence with medical treatment;
- Active treatment of infection anywhere in the body with IV antibiotics at screening and baseline;
- Suspected or confirmed signs/symptoms of gangrene on any part of the affected limb;
- Known osteomyelitis or bone infection of the affected foot or leg, as verified by diagnostic imaging within 30 days prior to enrollment;
- Subject is pregnant or breastfeeding;
- Study ulcer has a history of treatment with hyperbaric oxygen, growth factors or other biologic treatments, or a cellular or tissue-based product (CTP) within 30 days of enrollment;
- Subject has a known or suspected allergy to products under study;
- Concurrent disease or drugs known to induce severe photosensitivity of the skin, such as porphyria.

### 2.7. Study Procedures

At the screening visit (SV), Day 0, patients will be provided with an informed consent procedure and documentation of the informed consent discussion. The IRB-approved informed consent is obtained prior to performing any trial-related procedures. Once consent is obtained, the physician will document the onset of the ulcer per medical record or patient report, with preference to documentation over self-reporting. The patient’s medical history and demographic information will be recorded. For female patients of childbearing potential, urine or blood pregnancy tests will be conducted. A physical examination will follow and is focused on the integumentary system. Next, a Fitzpatrick score will be assigned. The Fitzpatrick score is a validated assessment of skin tones meant to increase enrollment of minority populations in clinical trials. This is necessary, as an objective assessment of skin color is recommended in all clinical trials, and when using optical diagnostics, the color of the skin is important data.

Due to the inaccuracy of length times width measurements using a hand-held ruler, wound surface area measurement will be assessed using digital planimetry. There are several systems commercially available that accurately measure wound surface area. In this trial, the MolecuLight device in standard photographic mode will provide accurate planimetry. Finally, the patient will complete a baseline pain assessment using a visual analog or numeric scale. Prior or concomitant medications will be reviewed. Quality of life questionnaires will not be used in this trial design due to the short duration of the trial.

This trial design does not incorporate a run-in period such as those used for advanced wound care modalities (e.g., cellular and tissue-based products for wound care (CTP). The goal of antiseptic trials is to identify products that reduce the level of bacteria in the wound; therefore, a run-in to assess healing rate prior to enrollment is unnecessary.

All consenting subjects meeting eligibility requirements will be immediately moved to treatment visit (TV) 1. Subjects will be seen by the principal investigator (PI) or designee on TV 1. After the first visit, the PI will prescribe the frequency of wound cleansing following the randomization scheme. In this trial, patients will be stratified by site and randomized using a block randomization scheme. Typically, a block randomization of four is used, with occasional blocks of two or six to prevent provider bias. Patients are randomized to either the treatment or control group using a 1:1 ratio. There are several commercially available automated systems that provide randomization services. They are convenient but add cost to the trial. The patient, PI, and study staff will remain blinded to the allocation.

Subjects will be seen weekly for a period of 28 days, +/−3, for a total of 5 visits. Table 1 details the schedule of events for the study.

The MolecuLight Procedure (MiX) will be completed using the MolecuLight i:X Imaging Device, a handheld point-of-care medical device comprised of a high-resolution color LCD display and a touch-sensitive screen with integrated optical and microelectronic components. MolecuLight i:X detects bacteria at levels greater than >10^4^ CFU/g [9]. The MiX procedure will be performed before and after wound cleansing for accurate point-of-care digital assessment of bacterial load on the target ulcer to aid the primary investigator or designee in the most precise wound bed preparation and monitoring of wound healing.

Following TV 5 or upon early termination for any reason, a study exit form will be completed along with placement of SOC dressings if the target ulcer is not healed.

Enrolled patients who meet the inclusion criteria and have provided consent will be randomized 1:1 to have their wound cleansed with either NSS-HG or AMC-AMG. Unblinded staff will prepare labeled solutions of the two treatments that are consistent with the randomization scheme. Unblinded staff will also provide the patient with a four-week supply of their assigned treatment.

After the screening visit, enrolled patients will start treatment visit 1. At this treatment visit, initial procedures will be conducted:

1. Confirmation of inclusion criteria and randomization to treatment;
2. Photography of the wound and digital surface area measurement;
3. Fluorescence imaging;
4. Wound cleansing with randomly assigned treatment;
5. Post debridement fluorescence imaging;
6. Swab of wound for protease activity;
7. Application of wound dressing at the discretion of the principle investigator;
8. A four-week supply of the assigned treatment is provided to patients;
9. If applicable, wound debridement, measurement, off-loading walker fitting (DFU applicable), or application of multilayer compression (VLU) is conducted.

At the subsequent treatment visits (visits two through four), all steps will be repeated except for step eight. At the end of the study visit (treatment visit five), final assessments will be conducted to review potential adverse events, concomitant medications, clinical signs and symptoms of infection, and pain status. Pain intensity of the reference ulcer will be assessed before and after any wound cleaning. If applicable, wound debridement, measurement, and photography will be performed. The study exit form will be provided to patients and additional placement of standard-of-care dressings will be provided if the wound has not healed.

Subjects will return weekly for 4 weeks after randomization unless the wound heals, the subject withdraws, or the subject is lost to follow-up.

### 2.8. Statistical Methods

Summary statistics including mean, median, interquartile range, counts, and percentages are used to summarize demographic and treatment data collected from patients.

When determining sample size, a power of 80% is needed to detect a difference between groups of 0.35. With sample sizes of 30 patients per group, 80% power is achieved.

A chi-square test of independence will be conducted to assess endpoints concerning proportions. For the BLEU trial, the chi-square test will be used to assess the primary and secondary endpoint. The primary endpoint is to determine the relationship between the treatment and the patient, reaching a threshold of 40% PAR in four weeks. The secondary endpoint is to determine the relationship between the treatment and a reduction of bacterial load of <10^4^ CFU/g (by Fluorescence) by the fifth treatment visit. The assumptions of the chi-square test of independence must be met. These include randomly selected patients, independent treatment groups, and the use of two categorical frequency variables. The expected frequency for each group must be greater than five. A significance level of 0.05 will be used for this hypothesis test. For the primary endpoint, the null hypothesis states that the proportion is equal for both treatments, whereas the alternative hypothesis states that the proportion is not equal for both treatments. Statistical software such as R (version 4.3.1), Excel (version 365), or a TI-83 calculator (TI-83 Plus Graphing Calculator|Texas Instruments) will be used to calculate the chi-square test statistic and the *p*-value.

A *t*-test will be used to evaluate endpoints concerning mean differences between treatment groups. For the BLEU trial, the t-test will be used to assess the secondary endpoint. The secondary endpoint is to determine the percentage wound area reduction at four weeks. The assumptions of the t-test must be met. These include randomly selected, independent, normal variables that have similar variance. A significance level of 0.05 will be used for this hypothesis test. For these endpoints, the null hypothesis states that the mean difference between the baseline and the end of the study is equal for both treatments, whereas the alternative hypothesis states that the mean between the baseline and the end of the study is not equal for both treatments. Statistical software such as R (version 4.3.1), Excel (version 365), or a TI-83 calculator (TI-83 Plus Graphing Calculator|Texas Instruments) will be used to calculate the test statistics and the *p*-value.

### 2.9. Subject Withdrawal

All subjects have the right to withdraw at any point during treatment without prejudice. Whether each subject completed the clinical study will be documented. If, for any subject, study treatment or observations are discontinued, the reason(s) will be recorded. The investigator can discontinue a subject at any time if it is considered medically necessary. The termination of study participation is not anticipated to affect subject safety. If a subject withdraws consent to participate in the study or is withdrawn by the investigator, attempts must be made to obtain permission to record at least survival data up to the protocol-described end of the subject follow-up period. Reasons for withdrawal or early termination will be obtained whenever possible.

A subject will be considered lost to follow-up if they cannot be contacted after 5 phone calls and 3 letters.

### 2.10. Subject Compensation

Patients will be compensated USD 35 after completion of each study visit. This is a nominal compensation that offsets the cost of travel, parking, and the additional time required to collect information specific to the study.

### 2.11. Anticipated Risks/Risk Mitigation

Anticipated risks associated with the study procedures are listed below, along with the applicable risk mitigation. Adverse events related to the treatment are unlikely. The potential risks are listed in Table 2.

## 3. Discussion

Chronic wounds are a growing public health problem [17]. A new medical specialty has arisen to meet the needs of millions of patients suffering from this disabling condition. As with any new specialty, well-conducted research into treatment regimens and diagnostics has lagged. The field of wound care needs standardized endpoints and clinical trial design. The Wound Care Collaborative Community was established by the United States Food and Drug Administration (FDA) in 2021. The goal of the organization is to standardize clinical trial endpoints, design, and technologies. In the past, the FDA only recognized complete wound healing as an approved endpoint for wound care clinical trials [11,12]. The WCCC has recognized that complete closure is not always the appropriate endpoint for wound care technologies [12]. For example, complete wound closure is not an appropriate endpoint for topical antiseptics that are designed to reduce bacterial burden and prepare the wound for technologies that facilitate complete closure. The WCCC has suggested several surrogate endpoints, such as a percent surface area reduction of 40% in four weeks [12]. The primary endpoint for the protocol described here employs this surrogate endpoint.

It is necessary to use double blinding for clinical trials involving antimicrobial agents. The benefits of double blinding in clinical research include detection of causality between the treatment and wound healing, reproducibility of the study, and bias reduction.

The BLEU protocol design incorporates the recognized surrogate endpoint of 40% PAR at 4 weeks. It is also important in antimicrobial trials to assess the reduction in bacterial levels. This is accomplished using fluorescence imaging, a point-of-care-validated device that eliminates the need for the invasive and costly biopsies needed for quantitative cultures. Unlike most wound-healing clinical trials, this design utilizes double blinding, which reduces bias.

The primary source of evidence for the use of antimicrobial cleansers and gels is lab and preclinical. The lack of clinical trials in the wound care population leaves many clinicians confused as to how and when to use topical antimicrobials for chronic wounds. There is also little guidance on the choice of antimicrobials. Utilizing the trial design described here will standardize clinical trials evaluating the efficacy of topical antimicrobials. It will also allow for comparative effectiveness studies between antimicrobials.

## 4. Conclusions

The BLEU clinical trial design to evaluate topical antimicrobials incorporates an accepted surrogate endpoint and assesses bacterial reduction in a blinded fashion. This trial design allows for the evaluation of topical antimicrobials in the chronic wound population.

## Figures and Tables

**Table 1:** Schedule of events.

	SV, TV 1	TV 2	TV 3	TV 4	TV 5/EOS
Window Period	Day 0	Day 7	Day 14	Day 21	Day 28
Informed consent	√				
Documentation of onset of wound	√				
Medical history and demographics	√				
Physical exam	√				
Urine or blood pregnancy test (females of childbearing potential)	√				
Infection status	√	√	√	√	√
Assessment of pain status—PEG	√	√	√	√	√
Prior and concomitant medication review	√	√	√	√	√
Wound cleansing	√	√	√	√	√
Wound measurements/photographs	√	√	√	√	√
Adverse event review		√	√	√	√
MolecuLightTM photos (2)	√	√	√	√	√
WoundChekTM protease test	√				√

**Table 2:** Anticipated risks.

Study Procedure	Anticipated Risks	Risk Mitigation
Wound cleansing	Pain, bleeding, allergic reaction (e.g., rash).	Procedures to be performed by trained clinical staff
Wound measurements	None anticipated	N/A
Pain assessments	None anticipated	N/A
Wound photos	None anticipated	N/A
BIAKOS™ Antimicrobial Skin & Wound Cleanser	Allergic reaction	Procedures to be performed by trained clinical staff
BIAKŌS Antimicrobial Wound Gel	Allergic reaction	Procedures to be performed by trained clinical staff
Dressing placement	None anticipated	N/A

## Data Availability

The data is proprietary, but is available on request to the corresponding author.
